# Treatment of Autism Spectrum Disorders by Mitochondrial-targeted Drug: Future of Neurological Diseases Therapeutics

**DOI:** 10.2174/1570159X21666221121095618

**Published:** 2023-04-12

**Authors:** Showkat Ul Nabi, Muneeb U. Rehman, Azher Arafah, Syed Taifa, Iqra Shafi Khan, Andleeb Khan, Summya Rashid, Fatimah Jan, Hilal Ahmad Wani, Sheikh Fayaz Ahmad

**Affiliations:** 1 Large Animal Diagnostic Laboratory, Department of Clinical Veterinary Medicine, Ethics & Jurisprudence, Faculty of Veterinary Sciences and Animal Husbandry, Sher-e-Kashmir University of Agricultural Sciences and Technology (SKUAST-K), Srinagar J&K, 190006, India;; 2 Department of Clinical Pharmacy, College of Pharmacy, King Saud University, Riyadh, Saudi Arabia;; 3 Department of Pharmacology and Toxicology, College of Pharmacy, Jazan University, Jazan, 45142, Saudi Arabia;; 4 Department of Pharmacology & Toxicology, College of Pharmacy, Prince Sattam Bin Abdulaziz University, P.O. Box 173, Al-Kharj, 11942, Saudi Arabia;; 5 Department of Pharmaceutical Sciences, CT University, Ludhiana, Ferozepur Road, Punjab, 142024, India;; 6 Department of Biochemistry, Government Degree College Sumbal, Bandipora, J&K, India;; 7 Department of Pharmacology and Toxicology, College of Pharmacy, King Saud University, Riyadh, 11451, Saudi Arabia

**Keywords:** Autism, autism spectrum disorders, mitochondria, mitochondrial dysfunction, therapeutic target, neurodevelopmental disorder

## Abstract

Autism is a neurodevelopmental disorder with a complex etiology that might involve environmental and genetic variables. Recently, some epidemiological studies conducted in various parts of the world have estimated a significant increase in the prevalence of autism, with 1 in every 59 children having some degree of autism. Since autism has been associated with other clinical abnormalities, there is every possibility that a sub-cellular component may be involved in the progression of autism. The organelle remains a focus based on mitochondria's functionality and metabolic role in cells. Furthermore, the mitochondrial genome is inherited maternally and has its DNA and organelle that remain actively involved during embryonic development; these characteristics have linked mitochondrial dysfunction to autism. Although rapid stride has been made in autism research, there are limited studies that have made particular emphasis on mitochondrial dysfunction and autism. Accumulating evidence from studies conducted at cellular and sub-cellular levels has indicated that mitochondrial dysfunction's role in autism is more than expected. The present review has attempted to describe the risk factors of autism, the role of mitochondria in the progression of the disease, oxidative damage as a trigger point to initiate mitochondrial damage, genetic determinants of the disease, possible pathogenic pathways and therapeutic regimen in vogue and the developmental stage. Furthermore, in the present review, an attempt has been made to include the novel therapeutic regimens under investigation at different clinical trial stages and their potential possibility to emerge as promising drugs against ASD.

## 1 INTRODUCTION

Mitochondria are the sub-cellular double-enveloped semi-autonomous organelles predominant in cellular function and energy production [1, 2]. Mitochondria, the powerhouse of cells, are predominantly found in skeletal and cardiac muscles, contributing more than 90% of ATP required for cell metabolism [3]. In addition to being an intracellular energy-producing organelle, mitochondria can also be present outside the cell, evoking either regenerative benefits or acting as a danger signal [4]. For the normal functioning of cells, mitochondria are required, and problems in the mechanisms that govern mitochondrial DNA (mtDNA) maintenance and function lead to disease development [1]. Mitochondria are involved in several cellular activities, like glucose, lipid and protein metabolism, and energy production, which control several signalling pathways.

Moreover, mitochondria act as nutritional sensors, and their biogenesis, dynamics, and interactions with other organelles are all dynamically controlled to modify metabolism [5]. Free, membrane-bound (in platelets and vesicles), and cell-free circulating mitochondrial DNA are the various forms of extracellular mitochondria that initiate various cellular and sub-cellular responses at different levels of metabolism. Stress, damage, or death of the cell leads to the release of mitochondria. Similarly, the healthy cells are actively released to recover the injured ones [6]. Mitochondria perform a unique role in cancer, ageing, and tissue injury by controlling various essential cellular activities [7]. Organs having higher metabolic rates (brain, heart, liver, and kidney) require optimal mitochondrial function [8]. To function correctly, neurons must produce adenosine triphosphate (ATP), exocytose synaptic vesicles, regulate intracellular calcium, and control neuronal membrane potential [9], and for these essential functions, they need mitochondria to supply energy in the form of ATP. Furthermore, neurotransmitter release, neural differentiation, and neuroplasticity are the other vital areas where mitochondria play a key role [10].

ASD is a diverse collection of neurodevelopmental illnesses, including autistic disorder, Asperger’s Syndrome, and pervasive developmental disorder. They are characteristically defined by social interaction and communication impairments with restrictive and repetitive behaviors during the early developmental period. In the United States, it is known to affect around 1.8% of children [11]. The etiology is not fully understood but most likely involves environmental factors affecting many vital processes in the body. It is worth mentioning that ASD is linked to predisposing factors, including aberrant redox and mitochondrial metabolism [12]. In reality, mitochondrial dysfunction seems prevalent in 5% to 80% of ASD children [12]. The majority of them demonstrate novel dysfunctions of mitochondria rather than a classic mitochondrial disease (MD) [13-15]. As mitochondria are vulnerable to environmental factors, the result makes the mitochondrion potentially a crucial mediator of genetic-environmental interactions [16].

## 2 MITOCHONDRIAL FUNCTION IN HEALTH

Mitochondria are essential for practically every aspect of disease progression [9]. Several studies reveal that adding extracellular mitochondria to the Cerebrospinal fluid (CSF) could be a viable treatment for neurodegenerative disorders, ischemia events, and traumas [2, 17]. Mitochondria in good working order play the main role in tissue regeneration. Apart from supplying ATPs for the energy needs of cells, mitochondria play many other important roles, like regulation of apoptosis [18] and immune responses [19]. Mitochondria are involved in the neurodegenerative process of Parkinson’s disease (PD) as impairment in their function leads to a defect in the electron transport chain (ETC).

The mitochondrion also regulates signal pathways for controlling viral infections [20]. Metabolic by-products of oxidative phosphorylation include reactive oxygen species (ROS), which disrupt the bio-molecular and cellular framework. External stimuli can trigger endogenous ROS, but the highest quantities of free radicals are formed in the mitochondria [21]. Several neurological illnesses are a result of mutations in mitochondrial proteins. A mutation in the mitochondrial genome at the site of 16S rRNA causes Dupuytren's disease, a maternally transmitted disorder characterized by abnormal thickening and tightening of the hands [22]. Mitochondrial disorders in humans are genetically transmitted and caused by abnormal mitochondrial DNA, affecting nearly every medical condition [23]. Mitochondrial mutations are inherited from the X chromosome or maternally, depending on where the gene abnormality is located. Currently, there is no specific cure for mitochondrial illnesses, and the only treatment available is to alleviate symptoms [24]. The most common genomic mutations encountered in mitochondrial disorders are located in gene loci encoding mitochondrial DNA polymerase, which can manifest in the form of diverse neuron-psychological dysfunctions [25]. The consequences of extracellular mitochondria and their fate within the recipient cell are still under study [26-28]. Recent research regarding the role of membrane/free, intact/damaged mitochondria in the inflammatory response and their interactions with polymorphonuclear cells in various models has found a prominent role being played by mitochondrial dysfunctions [29, 30]. In response to various signals, like cellular stress, injury, or in order to preserve the function of other cells, mitochondrial DNA is released as free mtDNA or packed into exosomes [31, 32]. Henceforth, the presence of increased concentrations of free mtDNA during chronic diseases acts as a prognosis and survival biomarker [28]. As mitochondria play a role in a variety of diseases, like Alzheimer's disease (AD), Parkinson's disease (PD), diabetes, acute myocardial infarction, and stroke, therapeutic intervention includes replenishment of the mitochondrial pool through artificial transfer [33, 34]. Accordingly, it has been observed that mitochondria change dynamics, kinetics, and gene expression to protect cells from apoptosis [35]. Based on these propositions, mitochondria are used to treat myocardial ischemia in order to promote tissue repair [36]. A standard therapeutic option nowadays is the transfer of extracellular mitochondria in cases of ischemia [37, 38]. Furthermore, recent studies have indicated that the transfer of mitochondria is the therapeutic choice in the case of nervous and cardiovascular system disorders [2, 39]. In order to maintain the healthy functioning of the cellular and sub-cellular framework and to prevent ageing process and age-related disorders, it is necessary that mitochondrial structure, function, and homeostasis should be maintained [40]. At sub-cellular levels, protective immunological, and tumoricidal differentiation of macrophages, and inflammasome activation are some important actions performed by mitochondria [41].

In the central nervous system (CNS), mitochondrial functions and health continue to be fervid. Cell survival or cell death is ultimately determined by the health of the mitochondria, since, without ATP, neurons strive hard to survive, which leads to cell death. Various diseases, like AD, PD, Huntington’s Disease (HD), and other neurological disorders have shown association with mitochondria and altered mitochondrial DNA [42].

The first piece of indirect evidence in favor of mitochondria's involvement in AD is the observation of impaired glucose and oxygen metabolism in the brains of patients. In addition to impaired oxygen consumption in patient brains, other experiments have observed decreased glucose metabolism, adding more proof of bioenergetic dysfunction and mitochondrial dysfunction in the disease [43]. Numerous descriptive investigations identifying structural and functional anomalies in these organelles have shown mitochondrial involvement in addition to clinical characteristics [44].

In PD, several genes indisputably relevant for mitochondrial homeostasis are linked to the disease. Some of the common genes are presynaptic protein, the E3 ubiquitin ligase Parkin, alpha-synuclein, PTEN-induced putative kinase 1 (PINK1), the protein deglycase DJ-1, ATPase 13A2 (ATP13A2), and vacuolar protein sorting-associated protein 35 (VPS35) [45].

Similarly, in HD, one of the many explanations that have been put forth refers back to mitochondria, proposing that medium spiny neurons, known for having very high energy demands, are particularly vulnerable to mitochondrial malfunction and respiration suppression caused by mutant Huntingtin [46]. Indeed, a number of mitochondrial abnormalities are seen in both disease-affected individuals and animal models, including reduced calcium buffering capability, compromised bioenergetics, increased oxidative stress, and aberrant trafficking and dynamics [47].

## 3 MITOCHONDRIAL DYSFUNCTION AND AUTISM SPECTRUM DISORDERS

### 3.1 Mitochondrial Overactivity

**Fig. (1):**
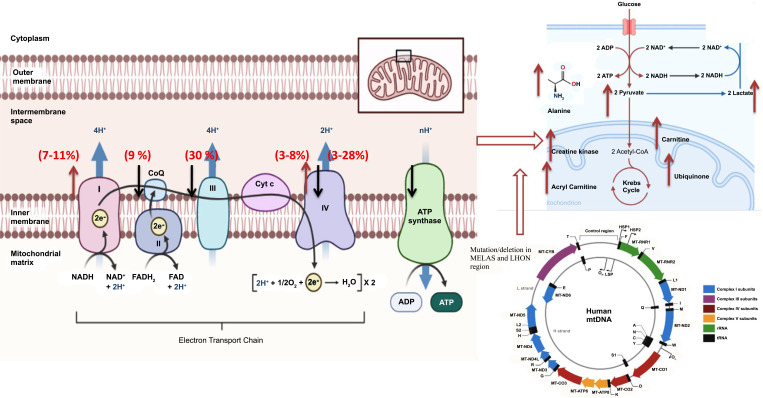
Major mitochondrial dysfunctions in children suffering from ASD with special emphasis on alteration in electron transport chain (ETC) and mutations/deletions in mt-DNA. Arrows pointed in the upper direction indicate increased activity while arrows pointed downwards indicate decreased functioning, and data in brackets indicate the contribution of various mitochondrial dysfunctions towards ASD. www.biorender.com

Mitochondria are involved in many cellular processes throughout the body; their dysfunction may cause problems in physiological functioning [9]. In addition to that, mitochondria are the principal generator of reactive oxygen species (ROS), and their levels get significantly increased when the electron transport chain (ETC) malfunctions [48]. It has been observed that ROS levels are excessively high in patients with ASD caused by mitochondrial malfunction compared to control [49]. Mitochondrial dysfunction has been diagnosed using diagnostic tools, including objective clinical, histological, biochemical, molecular, neuro-imaging, and enzymatic findings. Severe impairments in electron transport chain (ETC) complex activity are one of the hallmarks of classic mitochondrial diseases. Zussman *et al.* [17] proposed that ASD could be a condition triggered by reduced mitochondrial activity and altered brain bioenergetics. Following this, in the preceding two decades, the first evidence of mitochondrial malfunction in ASD has been proposed by various researchers across the world [50]. These studies have found that mitochondrial dysfunction affects up to 50% of people with ASD [51]. While a major population-based study found that 7.2% of children with ASD have mitochondrial disease, a controlled research study published in the Journal of the American Medical Association has reported that mitochondrial malfunctioning could affect as many as 80% of young ones with ASD [20]. As the proportion of young ones with ASD and concurrent findings of indications of mitochondrial abnormalities have become an established finding, in these ASD patients, slightly unique pattern of ETC has been explored. Noteworthy, among them, is the overactivity of the ETC complex, as it has been established that overactivity of ETC is the prominent finding in neurological damage occurring in a diverse set of diseases, as reported from multiple investigations. Graf *et al.* [52] were the first to notice the overactivity of the respiratory chain complex I and respiratory chain complex IV in a case of autism, and their work was the first to link autism to the overactivity of the mitochondria framework. To support this proposition, impaired mitochondrial function, microtubule energetics, and cell motility could all play a role in nervous system development. Hence, prenatal anomalies and autonomic dysfunction are two disorders associated with mitochondrial dysfunction. In line with this, ASD patients have revealed anomalies in brain growth and development [53]. One of the hallmarks of mitochondrial dysfunction in ASD is when the disease is associated with inflammatory events, gastrointestinal issues, seizures, motor delays, fatigue, and lethargy [30]. Physiological alterations, such as abnormal redox and mitochondrial metabolism, are linked to ASD. Mitochondrial dysfunction affects 5% to 80% of children with ASD, the majority of them exhibiting unique kinds of mitochondrial failure compared to traditional mitochondrial dysfunctions [25]. In a similar fashion, the majority of cases with ASD have a history of generalized epilepsy or subclinical epileptiform discharges on electroencephalogram with a history of developmental regression [54]. Similarly, all patients with ASD showed higher rates of exhaustion and lethargy, ataxia, GI issues, and normal brain imaging, and were less likely to have abnormal light microscopy [13, 14, 55]. A relationship between mitochondrial dysfunction and seizures in patients with ASD has been well established from clinical and pre-clinical studies [53, 55]. Similarly, Fillano and colleagues gave a detailed account of mitochondrial hyper-activity and ASD, and reported large-scale mtDNA deletions to be the prominent presentations of mitochondrial failure and ASD [56]. Furthermore, comprehensive research on over 3,400 ASD children in Taiwan found a substantial rise in the incidence of neurological illnesses, like epilepsy, peripheral nervous system disorders (*i.e*., neuropathy), and myopathies, as well as endocrine abnormalities. These studies attributed mitochondrial over-activity as the possible mechanism for the clinical manifestation of ASD [18, 57] (Fig. **1**).

Various prenatal factors, such as dietary agents, intrinsic and extrinsic stressors, commonly used pregnancy drugs, modulators of mitochondrial function, and potentially harmful genetic abnormalities for the fetus, are common risk factors for ASD and are all linked to mitochondrial dysfunction.

Gestational diabetes, a risk factor for ASD [58], has been linked to anomalies in carnitine metabolism [59]. Mitochondrial dysfunction is associated with increased oxidative stress and inflammation. In general, infection during pregnancy is a risk factor for ASD [60], and abnormalities in maternal trans-sulfuration metabolism and chronic oxidative stress are discovered in mothers of children with ASD during [61] and after [62] pregnancy.

Common nutritional deficits have been identified as prenatal ASD risk factors that may affect mitochondrial function. More severe ASD [63, 64] or an increased chance of ASD [65] are linked to decreased vitamin D levels during the first or second trimester as well as during the course of one's lifetime. Reduced mitochondrial respiration and oxidative stress are linked to vitamin D deficiency.

### 3.2 Biochemical Link

**Fig. (2):**
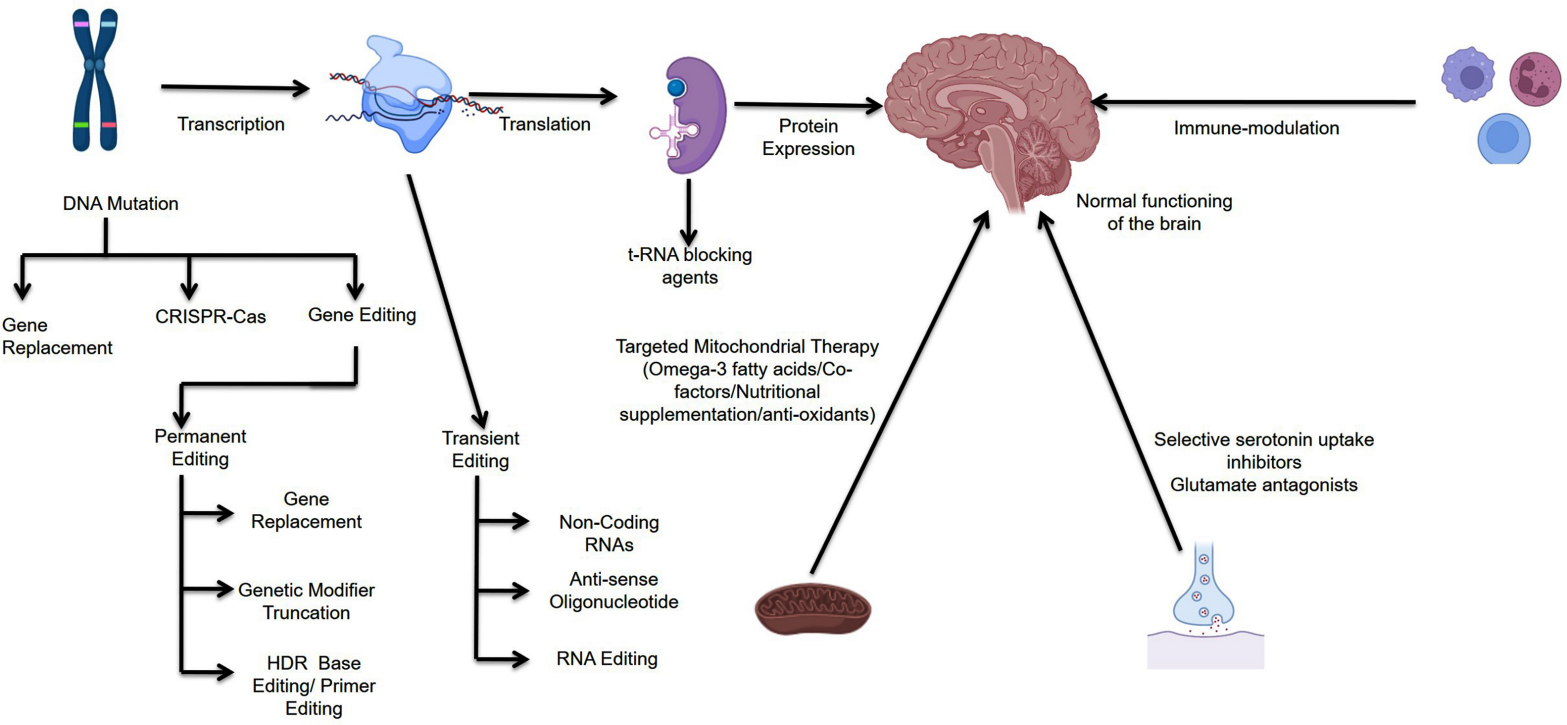
Possible therapeutic intervention for the treatment of ASD; the therapeutic intervention ranges from simple dietary changes to genome editing technology. There is enough possibility for these interventions to emerge as potential therapeutic regimens against the disease. www.biorender.com

The first report of the biochemical link in autism was reported by Coleman and Blass [66], who observed increased lactate levels in the plasma of four ASD patients in 1985, implying oxidative phosphorylation impairment. However, the role of mitochondrial disorder in autism was not presented until Haas established a clear link between these two dysfunctions [67]. The combined biochemical alterations, which include lactic acidosis, elevated levels of Krebs cycle metabolites, low plasma carnitine, as well as impaired glucose uptake by the brain, and low adenosine triphosphate levels, are all linked to autism, and these findings indicate the role of a biochemical link between ASD and metabolic alterations induced by mitochondrial dysfunction [68]. Over the last 30 years, a number of aberrant biomarkers have been observed in the brain and other biological fluids of children with ASD, corroborating the hypothesis of a bio-energetic deficit in ASD children [14, 68]. In concurrence with these findings, citric acid cycle metabolites were abnormal in the urine of patients with ASD, which is indicative of mitochondrial dysfunction [29, 35]. For diagnostic validation, none of these biomarkers were found to be specific for early diagnosis of mitochondrial dysfunction. In a meta-analytic study, various biochemical alterations have been observed in ASD patients, but the major problem with these biomarkers was the high degree of fluctuation of these markers [14, 69]. The degree of fluctuation can be gauged from six investigations with a number of samples ranging from 30 to 210 people, which compared lactate levels in ASD patients, and these studies found prevalence rates ranging from 17.1% to 76.6% [70, 71]. Furthermore, a controlled investigation identified mitochondrial malfunction in 80% of ASD children, despite that only 20% of these children had increased lactate levels, and only 10% had mitochondrial dysfunction [72]. Among all biochemical markers in ASD patients compared to contemporaneous controls, the important finding of interest was significant mean elevations in lactate and pyruvate levels and a significant reduction in carnitine and ubiquinone levels [14]. One of the important findings from these studies was an almost consistent lactate-to-pyruvate ratio, which ranged between 27.6 to 36.0 [14, 24]. Bantug and colleagues found significantly reduced levels of cerebral folate in three patients out of five patients who were tested for autistic disorder [41]. The link to cerebral folate deficiency was a significant finding in ASD patients because ASD symptoms were effectively reversed by folinic acid treatment/supplementation [73-75]. A comprehensive study was conducted earlier [76], and the authors found disturbances in fatty acid metabolism in people having ASD. The important findings of their study were alterations in the citric acid cycle and cholesterol metabolism, which resulted in elevated levels of unsaturated fatty acid metabolites. These studies attributed these anomalies to long-chain acyl-CoA dehydrogenase. Similarly, Frye reported metabolic defects in fatty acid oxidation, which were subsequently supported by other studies [15, 27, 77]. Furthermore, an examination of 213 ASD patients who underwent a metabolic test in a medically focused ASD clinic indicated that 17% exhibited persistent elevations in short and long acyl-carnitines (CESLAC), with C4OH, C14, and C16:1 statistically significantly higher than the upper limit of normal [54] (Figs. **1** and **2**).

#### 3.2.1 Direct Evidence from Brain Tissues

Researchers have found significantly reduced activity of complexes I and V and mitochondrial enzyme pyruvate dehydrogenase in various regions of the brain [67]. These defects were observed in 43% of autistic brains, while complex III impairment was identified in 29% [78]. Moreover, 29% of brain samples from autistic individuals revealed a mix of aberrant activity involving many complexes, while 14% indicated deficits across the board. The researchers also discovered an increase in the number of copies of mitochondrial genes [79]. In a study on post-mortem brain tissues, researchers found autistic developing brains to have reduced steady-state efficacy of enzymes of ETC in the frontal and temporal cortex. Furthermore, ASD children have significantly greater oxidative stress indicators in the cerebellum and temporal cortex, although relationships between these are yet to be established. These researchers postulated mitochondrial dysfunction in autistic children’s brains aged 4 to 10, indicating a potential vulnerability window [80-82]. In corroborating research, the Brodmann area 21 of the brain in patients with ASD was studied in post-mortem material and linked to autism phenotypic presentation, with the most striking finding being altered functioning of complex I and IV activity. The same authors also found decreased levels of anti-oxidant defense mechanism and, subsequently, more DNA damage [29]. In concurrence with these findings, studies identified significantly reduced protein levels of complexes I, III, IV, and V in the autistic brain. In addition to these findings, a greater degree of mitochondrial abnormalities was observed in children of the younger age group, which indicates a higher degree of vulnerability of the developing brain for ASD [76]. Another research examined the protein expression of 84 mitochondrial homeostasis genes in the post-mortem brain of individuals with ASD. Many genes were found to have reduced expression, such as MTX2 (mitochondrial pre-protein import receptor), NEFL (mitochondrial morphology, fusion, and motility regulation), and SLC25A27 (mitochondrial uncoupling protein 4) [83, 84]. The findings show that faulty mitochondria may play a function in autism that extends beyond oxidative phosphorylation. In this study, researchers discovered that autistic brains have lower protein expression of complex I, III, IV, and V subunits in the motor cortex, thalamus, and cingulate gyrus than controls. All brain areas investigated had decreased levels of ATP5A1 (complex V), ATP5G3 (complex V), and NDUFA5 (complex I) [83, 84].

#### 3.2.2 Indirect and Direct Evidence from Non-CNS Tissues

In a retrospective assessment of 25 children with autism's medical records, 76% had high blood lactate levels, 53% had increased pyruvate levels, 20% had increased lactate: pyruvate ratio in fibroblasts, and 42% had aberrant urine organic acid analyses [53]. A group of ASD patients was found to have lower total and free serum carnitine levels, lower pyruvate levels, and higher alanine and ammonia levels [85]; these anomalies are indicative of oxidative phosphorylation defects. In the skeletal muscle of children with autism, skeletal abnormalities were concurrently found in those patients who had mitochondrial defects, which ranged from defects in complex I, III, IV, and V [56]. Recently, various studies have found that children with autism have chromosome 15q11-q13 inverted duplication, which reduces skeletal muscle complex III activity [85]. Recently, a cohort study has found auto-immune antibodies against the mitochondrial M2 subunit in 52% of patients suffering from ASD. Furthermore, levels of these antibodies showed a positive correlation with the severity of the disease [86]. Similarly, a study conducted in Canada found significantly higher levels of polyunsaturated long-chain fatty acids in ASD patients compared to case-control patients [56]. When comparing patients with ASD to age-matched controls, lipofuscin deposits were detected in the frontal brain, presumably due to increased lipid peroxidation [87]. The mutations in the trimethyl lysine hydroxylase epsilon (TMLHE) gene, the products of this gene being the first enzyme in carnitine biosynthesis, have been linked to ASD [21, 88-91]. Furthermore, children with ASD in general [13, 92] and those with TMLHE mutations in particular [19] benefit from L-carnitine supplementation. Furthermore, decreased activity of complex I, complex III, and/or complex IV was detected in leukocytes or buccal mucosa obtained from autistic patients in a study published in 2010 [72, 93]. A number of further investigations in children with ASD have now validated these findings [94-97]. In a cohort study involving 60 autistic patients ranging in age from 2 to 40 years, 8.3% exhibited biochemical evidence of abnormal aerobic respiration [98]. Organic acids, like 3-methyl-glutaconic acid, citric acid cycle intermediates, and dicarboxylic acids were identified in the urine of ASD patients [67, 73]. In the preceding decade, some novel biomarkers have been identified, and these include lactate oxidase, pyruvate kinase, hexokinase [99] Na^+^/K^+^ ATPase [100-103], caspase 3 [101], and caspase 7 [98]. Identification of these novel markers can be beneficial for the effective targeting of ASD and can be beneficial in the development of a therapeutic regimen against ASD. In this direction, two more markers have been identified; these include growth differentiation factor 15 (GDF15) and fibroblast growth factor 21 (FGF21), both of which are newer mitochondrial biomarkers with strong specificity for MD [104].

#### 3.2.3 Immune Dysfunction and Inflammation

The initiation of inflammatory response and its effective resolution are essential for cellular and sub-cellular homeostasis to be maintained [35]. On the contrary, the disease can be caused by a weakened immune system combined with abnormal or insistent inflammation. Unchecked neuroinflammation in the brain, as well as its failure to resolve, can result in neuropathology and neurodegeneration [105]. Inflammation and immune system dysregulation have been identified in autistic brains, implicating their mechanistic significance [14]. The detection of persistent neuroinflammation in post-mortem brain tissues from individuals with ASD ranging in age from 4 to 45 years [40, 97] has been an important finding in ASD research [19, 40]. Inflammatory cytokines and chemokines production was observed to be increased in brain tissue and cerebral spinal fluid of patients with ASD [75, 92]. Microglia being nervous tissue residing in mononuclear phagocytic cells are involved in both immune surveillance and synaptic pruning during normal nervous development [106]. Hyperactivities of microglia have been observed in postmortem brain specimens of persons with multiple sclerosis, Alzheimer's disease, and Parkinson's disease, all of which have unclear genetic origins [107]. Multiple immune system-related genes have increased messenger RNA transcript levels in post-mortem brain tissue from ASD patients, showing that neuroinflammatory processes are involved in this illness [108]. Furthermore, a recent study of transcriptome organization patterns study found that gene co-expression networks in ASD patients’ brains imply aberrations in cortical patterning [19]. Many immune proteins present in plasma/serum, such as cytokines, chemokines, complement proteins, adhesion molecules, and growth factors, are significantly altered in ASD patients according to proteomic studies [109-115]. CCL2 and CCL5 levels in the blood are likewise higher in those with ASD, and they are linked to worsening behavioral scores [109]. Monocytes are among the first immune cells to respond to inflammation and are prolific cytokine makers, resulting in a cytokine environment that has a significant impact on the activity of nearby immune cells [101, 116]. ASD has been linked to an increase in circulating monocytes [72]. The unique pattern of cytokine responses was identified after TLR agonist stimulation of isolated CD14^+^ monocytes from young children with ASD in the lab [117]. Inflammatory cytokine production was increased in response to the TLR2 ligand lipoteichoic acid (LTA) and, to a lesser extent, the TLR4 ligand lipopolysaccharide (LPS). An unmethylated CpG repeat synthetic oligonucleotide that looks like bacterial DNA, on the other hand, reduced cytokine production. Higher monocyte IL-6 and IL-1b production in response to LPS stimulation has also been associated with social dysfunction in patients with ASD. Monocytes and perivascular macrophages have been found in higher numbers in the brains of persons with ASD, showing that hyperactivation of myeloid cells in ASD impacts both the peripheral and central nervous systems [85]. Furthermore, plasma cytokine levels in children with ASD who have a profile indicative of myeloid cell activation, such as IL-12p40, TNFα, IL-1β, and IL-6 production, are strikingly similar [109]. In patients with ASD, circulating T cell phenotypes had different activation profiles, with cytotoxic T cells displaying significantly elevated levels of HLA-DR (bio-marker of late cellular activation) [111]. Similarly, the killer T cell marker (dipeptidyl peptidase IV) found to be associated with CNS disorders, such as multiple sclerosis, was found to be increased [105]. T cells from children with ASD had a different pattern of co-stimulatory and activation markers following *in vitro* stimulation, with significantly increased expression of CD137 (4-1BB) but lower expression of CD134 (OX40) and CD25 (IL-2 receptor) [111].

#### 3.2.4 Oxidative Stress and Abnormal Redox Regulation

An imbalance between prooxidants and antioxidants causes reactive oxygen species (ROS) or reactive nitrogen species (RNS) to injure the cell [118]. ROS are signalling chemicals that can either endorse cell survival and tissue regeneration [119, 120] or suppress cell survival gene expression, hence causing cell death [120]. Distress is a potentially harmful oxidative stress that causes neuroinflammation and disrupts the astrocyte-neuron connection, leading to ASD [121-123]. The first reduction product of molecular oxygen is superoxide and a major generator of damaging free radicals, such as hydroperoxides and lipoperoxides. As a by-product of oxidative phosphorylation, complexes I and III produce superoxide, the most common free radical generated within mitochondria [104]. Low levels of reactive oxygen species (ROS) are required for physiological interaction and homeostasis, and they play a role in vascular tone modulation, erythropoietin production, and programmed cell death. ROS, on the other hand, can cause irreparable damage to DNA, cellular proteins, and membrane lipids when created in a pathogenic manner [26].

A number of neurodegenerative disorders have been linked to this kind of oxidative stress [124]. Excess ROS might be produced by the organism in response to pathogenic defenses, or it can be produced at normal levels but not neutralized due to the redox homeostasis system's limited antioxidant capacity. Patients with ASD have greater oxidative stress or redox regulatory abnormalities, implying that ROS plays a role in the autistic phenotype [67, 68]. These anomalies result in significantly increased oxidative damage to DNA, proteins, and lipids [29].

Furthermore, significantly lowered ratios of S-adenosyl-methionine-to-S-adenosylhomocysteine have been found in the plasma of children with ASD, indicating impaired methylation capacity and higher oxidative stress. Autistic children were shown to have greater amounts of the lipid peroxidation biomarker 8-isoprostane-F2 in their urine [26]. In a similar fashion, antioxidant enzymes have been found to be reduced in autistic people [28, 32]. In children with ASD, a link has been found between low levels of anti-oxidant enzymes and language loss [80]. Glutathione is a potent cellular antioxidant in the brain and is involved in a number of cellular/sub-cellular survival pathways [18]. Genetic polymorphisms in glutathione have been discovered in people with ASD [49, 125], and its level has been found to be associated with behavior [126]. Reduced glutathione (GSH) concentrations are lower, oxidized glutathione (GSSG) levels are higher, and the GSH/GSSG redox ratio is reduced in ASD patients as compared to the control group [127, 128]. Several lines of evidence suggest that oxidative stress and ASD are linked. First, in patients with ASD, reduced endogenous antioxidant capacity, lower total glutathione and cysteine levels, as well as reduced enzymatic activity of GPx, SOD, and CAT, have been demonstrated [76, 120]. The large declines in homocysteine, cystathionine, cysteine, and GSH levels suggest that the trans-sulfuration enzymatic pathway in children with ASD is most likely less efficient. The decrease in levels of these metabolites corresponds to a decrease in methionine levels [129]. The significantly higher levels of GSSG in people with ASD are corroborated by a research conducted earlier [49], which reported 72higher GSSG in children with ASD diagnoses compared to GSSG levels in neurotypical children. The unexpected changes in glutathione reductase (GR) activity in children with ASD diagnosis could be explained by the fact that GR activity is sufficient to maintain an expected GSH/GSSG ratio under physiological conditions when compared to levels in neurotypical children used as controls. Excessive intracellular oxidative stress, on the other hand, will cause GSSG to be exported to the plasma to restore intracellular redox balance [77]. Furthermore, there is a relationship between genetic predisposition and ASD diagnosis, suggesting that GST may have a role in oxidative stress and ASD risk [130].

Another study looked at metabolites in methionine pathways in ASD children diagnoses and discovered that the ratio of S-adenosylmethionine (SAM) and plasma methionine to S-adenosyl-homocysteine (SAH), a methylation capacity indicator, was significantly lower in ASD children diagnoses compared to neurotypical controls [49, 131]. Furthermore, the ASD group had significantly lower plasma levels of cysteine, GSH, and the ratio of GSH/GSSG, all of which are indications of antioxidant capability and redox balance [13, 132]. Higher levels of lipid peroxidation were found in those with ASD, suggesting that oxidative stress is causally linked to the diagnosis. Children with ASD experienced moderate to dramatic increases in isoprostane levels compared to neurotypical controls, resulting in lower phosphatidylethanolamine levels and greater phosphatidylserine levels. Reduced levels of anti-oxidant proteins have been linked to the loss of previously learned linguistic skills in children [127]. Abnormal methionine metabolism, high homocysteine levels, and oxidative stress have all been associated with neuropsychiatric disorders. Children with ASD had higher homocysteine levels, which were inversely related to GPx activity, lower human paraoxonase 1 arylesterase activity, and low vitamin B12 levels [133]. Owing to significantly lowered antioxidant capacity, increased demand for energy, and larger levels of lipids/ketones, nervous tissue is particularly prone to oxidative stress [134, 135]. Post-mortem analyses of the autistic brain have found aberrations in redox homeostasis enzymes as well as signs of oxidation to cellular biomolecules [14, 133]. In autistic patients, the temporal lobe had lower levels of anti-oxidant activity and significantly higher levels of 8-hydroxy-2′-deoxyguanosine, a hallmark of oxidatively damaged DNA [80, 136].

#### 3.2.5 Abnormal Calcium Homeostasis

Calcium plays an integral role in numerous cell signaling pathways [137]. Calcium signals are essential for synaptic transmission and for more complex brain activities. Changes in intracellular calcium levels have been important in nervous system disorders. Abnormal calcium homeostasis has been found to be associated with various neurological diseases [138]. In recent investigations, various calcium-dependent genes have also been linked to the etiology of autism spectrum disorders. Calcium has been shown to increase ATP production within mitochondria [139]. Calcium affects oxidative phosphorylation, and calcium buildup within mitochondria can cause the electrochemical proton gradient to collapse, resulting in bioenergetic failure and necrotic cell death [140]. Calcium causes mitochondria-mediated apoptotic cell death by increasing the permeability of the transition pore after binding to cardiolipin on the inner mitochondrial membrane [141]. Calcium homeostasis abnormalities can lead to mitochondrial malfunction, oxidative stress, and cytotoxicity [121]. Various genetic determinants that have been identified to be related to ASD and play a role in calcium homeostasis include the NMDA receptor subunit, kainate ionotropic glutamate receptor subunit 2 (GRIK2), or metabotropic glutamate receptor genes; mutations in these genes cause or result in the progression of ASD [85, 120, 142]. Autism has been linked to modulating the expression of genes involved in calcium signalling and homeostasis [14]. The calcium-dependent mitochondrial aspartate/glutamate carrier AGC1 and the gene that produces it (SLC25A12) are of particular interest because they link calcium regulatory problems to mitochondrial dysfunction [143]. Autistic individuals’ SLC25A12 expression was lower in the motor cortex and cingulate gyrus but higher in the prefrontal cortex [144].

Calcium is particularly important in neurons for action potential initiation and propagation. The events for signal transduction in neurons are widely regulated by intracellular calcium homeostasis. There are various calcium signalling pathways in the neurons. The inositol triphosphate (IP_3_) mediated calcium signalling is expressed in the ER membrane of all cell types. IP_3_Rs play a central integrating and a crucial role in neurons, as evidenced by the involvement of IP3R-mediated calcium release in synaptic plasticity and memory [145, 150], neuronal excitability [146, 147], neurotransmitter release [148], axon growth [149], and long-term changes in gene expression [146]. Skin-derived fibroblasts from individuals with the rare, monogenic variants of ASD fragile X (FXS) and tuberous sclerosis type 1 and type 2 were shown to have decreased IP3R-mediated calcium signaling (TSC) [151]. The neurological and behavioral phenotypes associated with autism are a result of abnormalities in IP3-mediated calcium signalling. However, there are a number of intricately linked processes involved in calcium transduction to the rest of the cell, and these pathways may be impacted by abnormalities originating from other sources. The pathogenetic mechanism by which they disrupt signaling is not known. Such abnormalities have been reported on ryanodine receptors (RyRs) and plasma membrane calcium ATPases (PMCAs) [151]. Gene mutations in RyRs have a limited role in ASD onset.

Similar to this, plasma membrane calcium ATPases have been mentioned as another source of aberrant calcium signaling in autism. Over the past two decades, a high co-occurrence of mitochondrial illnesses and autism has been thoroughly reported, pointing to a pathogenetic connection between the two conditions. Although the precise process by which the mitochondria produce the autism phenotype is still the subject of intense research, attention is shifting to the role that they play in calcium signalling.

### 3.3 The Genetic Link between Mitochondrial Dysfunction and Autism

The matrix of each mitochondrion contains several copies of the mitochondrial genome (mtDNA) [76]. mtDNA encodes 13 essential ETC enzyme subunits (complexes I, III, IV, and V), 22 transfer RNAs (tRNAs), and two forms of ribosomal RNA (rRNA). The remaining ETC complex components are encoded by nuclear deoxyribonucleic acid (nDNA). In addition, mitochondria include a variety of non-ETC enzymes, membrane proteins, and other molecular components necessary for mitochondrial activity and homeostasis [35, 76]. These proteins and enzymes are also encoded by nDNA [4]. As a result, genetic changes in either mtDNA or nDNA can cause mitochondrial dysfunction [79]. A genetic mutation is estimated to cause mitochondrial disease in one out of every 2000 children born in the United States. mtDNA mutations account for 15% of cases, while nDNA mutations account for 85% [152]. There are primary and secondary abnormalities in mitochondrial function. Gene mutations that limit aerobic ATP synthesis induce primary mitochondrial dysfunction, whereas oxidative phosphorylation deficits caused by other genetic or metabolic problems generate secondary mitochondrial dysfunction [67, 152]. Given the high prevalence of autism, it is logical to expect that some people with mitochondrial cytopathy will also be diagnosed with autism. One out of every 2000 children with ASD would be diagnosed with mitochondrial cytopathy if there was no relationship between mitochondrial malfunction and autism [152]. However, ASD cohorts have a higher prevalence of mitochondrial disease than the general population, showing that mitochondrial dysfunction plays a role in autism.

#### 3.3.1 Nuclear DNA Gene Mutations

Abnormalities in mitochondrial protein nuclear expression, as previously indicated, can jeopardize the functioning and ultra-framework of the mitochondria, and these alterations are responsible for the majority of inherited mitochondrial diseases [152]. Owing to these, researchers began to search for the relationship between autism and nDNA mutations; Filipek *et al*. described two examples of autism patients with an inverted duplication on chromosome 15q11-q13. Both children demonstrated mitochondrial dysfunction in skeletal muscle, with low complex III activity and considerable mitochondrial activity. The gene product may play a role in complex III regulation, despite the fact that the source of a 15q11-q13 anomaly is unknown [85]. In their analysis of a link between putative candidate genes in the 2q24-q33 area and autism, Ramoz and colleagues detected two single nucleotide polymorphisms (SNPs) in the SLC25A12 gene (rs2056202 and rs2292813) [153].

Furthermore, the calcium-dependent mitochondrial aspartate/glutamate carrier (AGC1) is encoded by the SLC25A12 gene, which has been connected to neurite formation. It has also been reported to be elevated in autistic children's prefrontal cortex [144]. These SNPs in the SLC25A12 gene were found in 48% of autistic patients [153]. Although other researchers have corroborated comparable findings, it is important to note that certain studies have yielded inconsistent results [143, 154, 155].

Other areas of nuclear DNA have been identified to play a role in the autistic phenotype. For instance, Pons *et al*. [156] studied the 7q32 region and detected two SNPs associated with ASD in the NDUFA5 gene (rs12666974 and rs23779262). NDUFA5 codes for NADH–ubiquinone oxidoreductase 1 alpha subcomplex 5. As a result, such changes could have an effect on complex I activity. According to other research, small cohorts of studies have found 1 Mb deletion in the 5q14.3 region [93], which resulted in a significant reduction of complex IV activity, and complex I activity was only slightly reduced. According to the authors, proteins produced from the likely gene influence the polymerization and functioning of complexes I and IV subunits of ETC. In a recently concluded study, there was an increased incidence of genetic aberrations in autistic children with mitochondrial disease [14]. This demonstrated that children with ASD and mitochondrial disease were part of a distinct group. Only 21% of the individuals in this sample exhibited mtDNA or nDNA mutations or chromosomal abnormalities, according to the researchers. As a result, the majority of cases were not linked to known genetic illnesses, suggesting that secondary mitochondrial dysfunction may play a role [14] (Table **1**).

#### 3.3.2 Mitochondrial DNA Mutations

Co-incidence of ASD and mitochondrial disease has been reported in various studies conducted across the globe; a significant finding was the prevalent occurrence of mtDNA flaws [67, 68]. The significantly higher proportions of these mitochondrial diseases and mutations are well-known and categorized, but others are less well-known and have yet to be recognized [56]. Despite the fact that this group of patients did not fit into any of the previously defined mitochondrial sickness categories, they showed autistic symptoms and 50% had large-scale mtDNA deletions [56].

In general, such observations have the disadvantage of being unable to make causal inferences. As a result, many of these studies can only conclude that mtDNA mutations cause autism. However, some data suggest that mitochondrial dysfunction may contribute to the autistic phenotype [55]. One of the most well-known mitochondrial disorders, mitochondrial encephalomyopathy, lactic acidosis, and stroke-like events (MELAS), has been related to autism. MELAS is caused by the A3243G mtDNA mutation, which has also been related to autism [157, 158]. These studies established a direct relationship between infantile encephalopathy, usually caused by MELAS A3243G mutation, and the development of autism in later life. Sue *et al.* then investigated five autistic children with familial history of mitochondrial disease, and all of these subjects were found to have mutations at three levels, which include A3243G and A3243G mutation in nuclear DNA and mtDNA depletion syndrome [157].

In another study, point mutation (G8362A) in the mitochondrial tRNA for lysine was identified as a possible alteration that causes the progression of ASD [159]. Another study examined the medical records of 25 persons diagnosed with ASD as their primary diagnosis. Among them, twenty-one had a mitochondrial disease that was confirmed, while four others had a mitochondrial disease that was suspected. Subsequently, a genomic-wide association study (GWAS) was conducted, and mtDNA mutations were observed in seven cases; the most commonly affected region included ND1 and ND4 complex I subunits, as well as the mitochondrial tRNA loci, in the mitochondrial genome [53, 160]. The CHARGE (Childhood Autism Risk from Genes and Environment) study was conducted on leukocytes of ten children with ASD aged 2 to 5 years [161]. They found larger mtDNA copy counts in ASD patients than in controls, and 2 of 10 autistic children had cytochrome b gene alterations linked to excessive replication.

A comparative study was conducted on 67 autistic children and 46 controls were taken from a group of patients from the CHARGE study. The researchers examined mtDNA mutations in the CYTB and ND4 genes, which code for cytochrome b and the ND4 subunit of complex I, respectively. They found that children with autism had a considerably higher rate of these mutations than children who did not have autism. In comparison to 7% and 9% of healthy children, ASD patients had an ND4 deletion and a CYTB mutation in 15% and 21% of cases, respectively [113, 161]. According to full exome sequencing from 903 ASD proband-mother-sibling trios, autistic children were 53% more likely than unaffected siblings to have heteroplasmic mutations in non-polymorphic sites (regions more likely to cause negative effects in oxidative phosphorylation). Non-autistic siblings had 1.5 times as many non-synonymous mutations and 2.2 times as many potentially damaging variants as autistic siblings [134]. When the proband's father was employed as a control, a recent study that sequenced the full mitochondrial genome of 400 autistic children failed to establish evidence of a relationship between mitochondrial defects and autism [162]. Recent research has used the real-time polymerase chain reaction (q-PCR) method to look at mitochondrial DNA copy numbers in persons with ASD. A structural variation in DNA that produces abnormal variation in the number of copies of one or more sections is known as DNA copy number variation. In their work, Gu *et al*. [79] identified insufficient ETC complexes and decreased pyruvate dehydrogenase activity. Variants in mitochondrial respiratory chain gene copy numbers were also investigated (CNVs). CNVs are the most common structural changes in the human genome. Increased CNVs were discovered in three respiratory chain genes that encode complex I and III subunits, a finding that has previously been observed in the lymphocytes of ASD patients in a study by Giulivi *et al.* [51]. Bons *et al*. [124] conducted a more recent study and found comparable results [96]. According to the study, the number of copies of mtDNA in peripheral blood cells is substantially higher in children with autism [126] (Table **1** and Fig. **1**).

## 4 MITOCHONDRIA DRUG TARGETING RATIONALE FOR AUTISM SPECTRUM DISORDERS

### 4.1 Targeting Mitochondrial Dysfunction

#### 4.1.1 Co-factor Supplementation

The use of co-factor supplementation in a wide spectrum of mitochondrial disorders has been well documented by researchers from different parts of the world [1, 7, 68, 164]. The popularity of these agents in mitochondrial disorder may be attributed to peculiar characteristics, which include these substances being well tolerated by the body and no short-term or long-term side effects being reported [40]. Most of the co-factors used against mitochondrial disorders belong to the vitamin B complex family, which being water-soluble are excreted through urine, and hence, there are no reports of bio-accumulation [125, 164]. Table **2** summarizes the most common co-factors used for mitochondrial disorders. Vitamin B complexes have been used against ASD, and very encouraging results have been reported from researchers across the world. Among different co-factors used for amelioration of ASD, L-Carnitine has been most widely used, and patients under L-Carnitine treatment have been reported to have significant improvement in symptoms compared to patients in the control group [159]. Recently, many studies have used L-Carnitine in double-blinded clinical trials; these studies have reported significant improvement in clinical scores of patients under the L-Carnitine arm compared to patients under the control arm [53]. Furthermore, these studies reported a positive correlation between L-Carnitine levels in the blood of patients and clinical improvement observed in ASD patients. The only side effect so far reported with chronic use of L-Carnitine includes loose odorous stool in a limited number of patients. Recently, a case-controlled clinical study has reported the therapeutic benefits of L-Carnitine in patients with lethal mutations in the Carnitine pathway [49]. Henceforth, clinicians can add L-Carnitine as substituent therapy with the principal drugs, like valproic acid, for accelerated recovery of patients suffering from ASD. The therapeutic efficacy of co-factor supplementation has been attributed to improvement in antioxidant potential [13], enhancing the efficacy of the folate pathway, increased ETC Complex I [53], and citrate synthase activity [57]. The therapeutic activity of different co-factors upon supplementation has been described in Table **2**.

#### 4.1.2 The Ketogenic Diet

The ketogenic diet has been evaluated in ASD; diet is clinically more important as KD has been successful in the resolution of ASD symptoms in patients not showing any improvement with commercial drugs used to treat ASD [16]. The efficacy of KD is based on the historical observation of anti-seizure effects of fasting and starvation in ASD patients; although metabolic effects of KD involve increased production of ketone bodies and decreased production of carbohydrates, researchers have attributed the clinical recovery to increased levels of polyunsaturated fatty acid as these bio-molecules dampen neuronal excitability [163]. KD has been found to be therapeutically safe in patients suffering from an epileptic form of ASD; in clinical settings, KD has been found to be superior in controlling seizures and ameliorating core and related symptoms of AS compared to conventionally used anti-epileptic drugs. A modified form of the KD, known as Atkins Modified Diet (AMD), is nowadays used against ASD, with the diet being less restrictive compared to conventional KD [164]. Over a period of time, KD has been evaluated in 2 uncontrolled and 1 placebo-controlled clinical trial, while AMD has been evaluated in 1 clinically controlled open-labeled clinical trial for its efficacy against ASD [165]. All these studies have reported significantly higher clinical improvement of ASD symptoms in the intervention group compared to the control group, and only the AMD study reported side effects of dietary intervention, and that also in the early stages of intervention. These studies attempted to highlight the therapeutic role of dietary intervention by identification of the pathogenic hotspots involved in disease pathways; hence, they evaluated levels of biomarkers in the intervention group and found that amelioration of the symptoms may be attributed to significantly lower levels of high-density lipoprotein, ornithine, acetoacetate, cesium, *N*-acetyl-serotonin and albumin in patients under dietary intervention group, and significantly increased levels of chromium and creatine across the treatment period correlated with better outcomes [166]. Pre-clinical studies in environmentally and genetically induced rat models of ASD have shown encouraging results; more pronounced improvement was observed in sociability and repetitive behavior of female rats of environmentally induced ASD with maternal immune activation (MIA)-induced ASD and mice with prenatal valproic acid exposure-induced ASD [167]. These pre-clinical studies attributed the recovery in symptom profile to modulation of glucose transport, over-expression of mitochondrial genes, bypassing defective pathways of ETC (electron transport chain), and improvement in mitochondrial biosynthesis, modulation of microbiota, regulation of brain neuronal networks, and promoting synthesis of white matter in the brain [168]. Although from a clinical setting, it is evident that KD is effective in the amelioration of cognitive and sociability symptoms in ASD patients, a clinically controlled randomized placebo study is lacking, which decreases its validity [169]. Furthermore, in children suffering from ASD because of the deficiency of pyruvate dehydrogenase complex, supplementation of KD has resulted in improvement in ataxia, sleep disorders, social behavior, and other related neurological functions. In addition to this, KD has been found to dampen the clinical symptoms in ASD patients having genetic mutations except for mutations of SUCLA2 or BOLA3. Researchers and clinicians have found KD as a promising therapy, but there is an urgent need to conduct clinically controlled, double-blind, randomized clinical trials [19, 170]. The major limiting factor of KD has been identified as tolerability in some patients, which can be modulated in further studies. Another limiting factor includes the implementation of dietary therapy, which many families find very tedious to implement, so families should be counseled before implementing dietary therapy in order to obtain desired results [96, 171] (Table **2**).

The hallmark therapeutic activity of KD in ASD involves the production of significantly higher levels of ATP through bio-energetic pathways involving oxidation of NADH, dampening of inflammatory pathways, maintaining the integrity of the mitochondrial membrane, *de novo* biogenesis of mitochondria, reduction in the activity of pro-apoptosis factors, and reduced production of ROS. Similarly, some authors have reported that the use of KD results in the reduction of pro-inflammatory cytokine levels, with the most prominent among them being IL-1 and TNF-α. Recently, some studies have reported that KD causes increased biogenesis of adenosine, which is involved in neuroprotection and neuron-modulator pleiotropic role [73, 113, 172]. To further support this proposition, adenosine, when supplemented separately, results in the amelioration of co-morbidities associated with ASD. Owing to these beneficial metabolic mechanisms of KD, it can be well speculated that dietary modifications are involved in the regulation of synaptic functioning and morphology. In addition, dietary modifications have been found to be involved in the modulation of neuronal excitability through action on potassium ion channels, neuron-transmitter transmission, synaptic neuron-transmitter cycling, hippocampal mossy fiber sprouting, and possibly through increased production of GABA [173].

##### 4.1.2.1 Other Metabolic Targets

Researchers across the world have reported the beneficial effects of various mineral/vitamin/other preparations in amelioration of clinical presentation in ASD and in the biochemical manifestation of the disease. The use of a wide spectrum of medicinal and dietary preparations has been used for the management of behavioral symptoms because the underlying cause is not known [19]. Efficacies of these interventions seem to be effective if these interventions are implemented in the early phases of the disease; however, accumulating evidence on the use of these interventions has provided insight into the probable mechanisms and pathogenic pathways of the disease, which can be used for the development of novel therapeutic regimens for the treatment of the disease. Supplementation of anti-oxidant preparations, which include vitamin C, N-acetyl-L-cysteine, *α*-tocopherol, methylcobalamin, and carnosine, has been widely evaluated in both pre-clinical and clinical studies, and encouraging results have been obtained from these studies [96]. All these formulations were found to cause a significant increase in levels of anti-oxidant defense mechanism, methylation of erythrocytes, and increase in ATP, sulfation, and increase nicotinic intermediates. Similarly, various herbal remedies have been evaluated in ASD, and they have yielded quite interesting results [174]. Recent studies have found a direct relationship between foliate deficiency and the clinical manifestation of ASD in children. Mitochondrial dysfunction and the production of auto-antibodies against foliate-α receptors cause deficiency of foliate, which culminates in ASD. Similarly, in laboratory animal models, severe foliate deficiency resulted in a significant reduction in cerebral folate levels, which subsequently manifested into ASD behavioral disorders [164, 175]. To further validate these hypotheses, experimental animals having significantly higher levels of foliate-α receptors on supplementation with folinic acid resulted in significant amelioration of communication skills, cognitive skills, sleep disorders, and other stereotypic symptoms of ASD. Currently, there is no available treatment for ASD, as pathogenic pathways and risk factors of the disease are yet to be established completely; henceforth, treatment options are limited to those agents that ameliorate the clinical presentation of the disease [176]. However, there are limited clinical studies that have reported the role of metabolic therapies in the amelioration of stereotypic symptoms in ASD. But all the metabolic studies have found significant clinical improvement in more than 50% of the participants under metabolic studies, while the remaining participants have shown minor improvements [177]. At present, there is an urgent need for a clinically controlled large-scale clinical trial on the role of metabolic therapy in the amelioration of ASD symptoms in order to obtain a clear picture of targeting pathogenic hotspots of the disease. As there is every chance that metabolic therapy may be beneficial only in the sub-group of the ASD population, thus it is required to establish the relationship between metabolic therapy and the genomic profile of ASD patients [178].

##### 4.1.2.2 Evidence from Animal Models

Owing to the complexity of ASD, researchers have employed diverse animal models to evaluate the efficacy of metabolic therapy. Some of these models have found a clear underpinning between metabolic disruption and symptoms of autism. In an animal model with succinic semialdehyde dehydrogenase (SSADH) deficiency, mimicking ASD in terms of symptomology metabolic therapy resulted in the normalization of EEG (electroencephalogram) and restoration of neuronal current in the hippocampus [174-178]. Another genetic model that resembles ASD is the Rett syndrome animal model; in this disorder, there is normal neuronal development in the early stages, but slow development of neurons and neuronal networks in the later stage of growth occurs. Normally, the disease is caused by a deficiency of methyl-CpG-binding protein 2 (*MECP2*) genes; studies involving animal models of Rett syndrome reported beneficial effects of metabolic therapy. BTBR T+tf/J (BTBR) inbred mouse strain is the clinically most relevant model for the study of ASD, as the animal model exhibits all core characteristics of the disease. Henceforth, these animal models have been used to study disease pathogenesis and evaluate novel therapeutic interventions against ASD [176]. These animal models showed improvement in clinical scores after feeding KD and in biomarkers of the disease. Recently, an interesting finding has been published; according to it, the gut microbiota of ASD patients varies significantly compared to clinically normal individuals. To further support this hypothesis, the researcher has found that metabolic therapy causes alteration in gut microbiota, which may be involved in targeting potential hotspot of ASD [162]. The most striking feature observed by these studies was the increased Firmicutes to Bacteroidetes ratio, which is mostly reduced in patients with ASD. Related to these propositions, it has been observed that dietary glycemic index has a direct relationship with the clinical manifestation of ASD. Based on the laboratory model studies, it may not be inappropriate to postulate that dietary/metabolic therapy alters the expression of genomic determinants of ASD. Taken together, results from preclinical studies have indicated the beneficial effects of dietary/metabolic therapy in the management of ASD [175-178]. However, clear evidence to establish a mechanistically prominent pathway remains yet to be established, as a limited number of studies to establish mechanistic pathways have been conducted on laboratory animals and humans. Thus, further studies on a diverse set of *in silico, in vitro,* and *in vivo* animal models are required and standardization of the pathognomic behavioral assay in order to establish pathogenic hotspot of disease and to evaluate the novel dietary/metabolic therapy that can provide insight into the neurobiology of ASD is warranted. Borrowing from the rich literature on encouraging results obtained from dietary/metabolic therapy in improving the efficacy of mitochondrial functioning, dietary/metabolic therapy can provide promising candidates for further studies [176].

### 4.3 CRISPR/Nano-CRISPR

Technologies and treatments that rely on editing genomic determinants of the brain may revolutionize therapeutic protocols and can help in better understanding the disease. Various studies have used intracranial injections of CRISPR-Gold as cargo to deliver CRISPR-Cas9 ribonucleoprotein that can edit genes in almost all cells of the brain, with no side effects so far reported. The CRISPR-Gold NPs effectively ameliorated the clinical signs of repetitive behavior and normalized the biomarkers characteristic of the disease [179]. CRISPR-Cas editing of the brain offers an important advantage of targeting localized genomic determinants, sparing systemic neuronal toxic effects. Furthermore, this novel technique causes a permanent change in the neurological framework, hence making repeated injections unnecessary andenhancing its utility in diverse clinical settings/disorders. Several genes have been identified that are potential targets of CRISPR-Cas therapy. Of special interest is the metabotropic glutamate receptor 5 (mGluR5) gene because this gene has been found to be over-expressed in ASD; following this, various pharmaceutical companies have started to search for small compounds that can specially target the mGluR5 gene. The search for these molecules was exhausted, as non-significant results were obtained from clinical trials using these small compounds [180]. Hence, knocking out the mGluR5 gene is hypothesized as a potential therapy to treat the clinical and biochemical manifestation of ASD. However, there are several limitations associated with this novel intervention, which need to be addressed before this intervention can be used in actual clinical settings. Identification of the effective vehicle for delivery of Cas9 ribonucleoprotein and identification of the brain region that needs genomic editing need to be carried out [181]. Although, to some extent, these limitations are addressed by CRISPR–Gold NPs, there is a need for further standardization and evaluation of these NPs in case-controlled randomized clinical trials. CRISPR-Cas metal NPs have a very wide cell tropism and have the potential to edit multiple sets of cells present in the brain; this is beneficial as in a large number of neurological diseases, a wide spectrum of cells are involved. Though these characteristics are advantageous, there is an urgent need to develop therapeutic preparation based on CRISPR-Cas, which can effectively target particular cell types [182]. Taken together, metal-based CRISPR-Cas NPs for gene editing can ameliorate most of the clinical and biochemical dysfunctions in ASD patients. Recently, researchers have developed a CRISPR-Cas-based technique for the treatment of Rett syndrome, a disease closely associated with ASD. The researcher evaluated this novel approach using fluorescence genes and found this approach as safe and effective in laboratory animal models; after three months of intervention, the expression of the target gene was decreased to half of its prior treatment expression. Similarly, a recent study conducted by Yip in laboratory animals using RNA-guided endonucleases Cas9 and Cpf1 using metal-coated CRISPR directed towards mGluR5 gene resulted in amelioration of repetitive behaviors of autism caused due to genomic defect of fragile X syndrome [183, 184]. Based on the results of these studies, researchers have postulated that CRISPR-Gold technology may be utilized for other neurological conditions, for instance, Huntington’s disease, in which polygenic determinants are involved. Owing to the fluorescence emissivity and functionalization of metal NPs, these can be used for real-time observation of the pharmacological effects of cargo while editing genes. Taken together, metal-based cargos of CRISPR-Cas can open novel ventures for delivering gene-editing tools that can serve as potential therapeutic technology to be used in actual clinical settings [183-187] (Fig. **2**).

### 4.4 Personalized Mitochondrial Medicine

Despite the search conducted by researchers, it has been very tedious for researchers to identify the disease in early childhood when therapeutic interventions are effective. One of the major limitations in the effective management of the disease is the heterogeneous presentation of clinical signs and heterogeneous genomic framework; hence, as pathways of disease vary in different individuals and different co-morbidities are present in different individuals, the response to treatment also varies across the patients [188]. Thus, researchers have postulated personalized medicine approaches for individuals with ASD owing to their specific and unique characteristics. Regardless of the various approaches utilized by different researchers, the majority of these studies have concluded that patients with ASD are unique in many ways, and hence, require a personalized approach for the amelioration of ASD symptoms [189]. In this section, we have attempted to describe the unique mitochondrial dysfunctions in ASD patients and hence their potential targeting using precision personalized mitochondrial medicine. A recent study [3,189] has found that there exists unique mitochondrial dysfunction in patients with ASD; in continuation, this researcher has found significantly elevated levels of acyl-carnitines in a subtype of ASD patients, which indicates changes in the β-oxidation pathway and electron transport chain in a subset of these patients, thereby leading to differential presentation of disease in different patients. Similarly, other studies have found neurotransmitter dysregulation associated with mitochondrial disease in a subset of the ASD population [190]. To support these propositions, some studies have found other mitochondrial dysfunctions responsible for the differential presentation of disease in patients with ASD. For instance, in some children with ASD, due to mitochondrial dysfunction, cognitive networks cannot be expressed, causing locked-in network syndrome. Based on these studies, we believe that there is an individual difference between clinical manifestations/presentation, pathogenesis pathways involved, and hence treatment options adopted [158]. Apparently, these studies highlight the importance of mitochondrial dysfunction as a potential determinant in the differential presentation of disease in different individuals. Although the relationship between ASD and mitochondria is not new, the unique correlation varies across the different patients suffering from ASD, and an in-depth understanding of this correlation can help in better targeting of disease, and hence, the development of personalized mitochondrial therapy in ASD patients [191]. Another important factor involved in the pathogenesis of ASD includes the impact of the immune system in which various factors play a role, and these factors have individual variations. For instance, cells involved in the immune response are dependent on energy supply, and due to mitochondrial variations, energy supply varies across different individuals [120]. In concurrence with this, foliate transport in the brain depends on the energy supply provided by mitochondria, so variations in mitochondrial functioning influence foliate levels. The “bad trio” involved in the pathogenesis of ASD includes dysfunctioning in mitochondria, redox imbalance, and immune dysregulation, although other factors are directly or indirectly related to this bad trio. In addition to these individual variations, gut microbiota produces metabolites that vary across the population [192]. These metabolites interact with mitochondrial metabolism; hence, these concepts form an important research area for further exploration to develop personalized mitochondrial medicine for ASD patients [128].

### 4.5 Gene Therapy in Autism

Researchers categorize ASD under monogenic disorders; a majority of researchers working in this direction have identified single-gene mutations as the probable cause of the disease. Intensive genomic studies have confirmed that ASD has a strong genetic basis; for this reason, the disease is a promising candidate for gene therapy [193]. All these studies have found that syndromic ASD is caused by a loss in protein functioning, which indicates that therapies should be directed toward the production of healthy gene products and, subsequently, their stabilization to compensate effects of mutation. Although these studies of gene editing for ASD have been conducted in pre-clinical stages and encouraging results have been obtained [194]. There are two main categories of gene editing: (i) those techniques that do not change the host genome, and (ii) those techniques that cause permanent changes in the host genome. Therapies that do not cause any change in the host genome include the use of non-coding RNA (nc-RNA) antisense oligonucleotides (ASO), RNA editing, and gene delivery. Non-coding RNA (nc-RNA) involves the use of small molecules of RNAs, which act on mRNA through “RNA interference pathways” [132]. These molecules inhibit the expression of those pathways involved in the expression of molecules manifested as the clinical presentation of the disease. For instance, factors that are involved in inhibiting the expression of target genes can be inhibited by the use of RNAi (RNA interference); henceforth, the expression of a target gene can be enhanced. For example, the target gene *SCN1A* expression was found to have a negative correlation with *RACK1* and *MDH2* gene expression [195]. Henceforth, inhibition of RACK1 and *MDH2* genes was found to cause up-regulation of *SCN1A genes.* Unfortunately, very little is known about the regulation of these genomic determinants and the associated side effects of interventions targeting these genes. Small activating RNAs (saRNAs) are novel molecules aimed to enhance the gene expression of target genes by acting at the promoter region. Circular RNAs (circRNA) are molecular adhesives that bind to inhibitory mi-RNAs, and hence enhance the expression of target genes at genomic levels [152]. Similarly, SINEUPs are novel interventions designed to bind with the non-coding region of m-RNAs, hence they prevent these molecules from degradation, increasing their half-life and enhancing the production of proteins that work on disease pathways. Another class of molecules used in the treatment of ASD includes antisense oligonucleotides (ASO); these molecules are noncoding/single-stranded genetic materials (DNA, RNA, DNA-RNA hybrids), and these molecules bind with mRNA molecules transcribed from lethal genes, and hence, inhibit the expression of these pathological mRNAs. Recently, ASOs have been used to affect pre-mRNA splicing, which leads to the synthesis of non-functional proteins. Finally, gene delivery is the recent therapeutic intervention that involves injecting extrachromosomal DNA (ecDNA) into the host cell without causing alteration in the host genome [156]. Furthermore, programmable RNA editing technology is gaining the attention of researchers as the procedure involves editing the genome at the transcriptome level. The non-genome editing technologies do not alter the host genome, which necessitates their repeated usage while keeping a check on the adverse effects, but the immune response to repeated use poses a concern for researchers and clinicians [158] (Fig. **2**).

The second category of gene therapy includes all those therapeutic interventions that cause a permanent change in the host genome; there are three main therapeutic interventions under this category, which include gene replacement, gene editing, and CRISPR-KO [154]. Gene replacement involves the introduction of cDNA, which gets integrated into the host genome and serves as a replacement for disease-causing mutational genes. Gene replacement involves the injection of lentivirus cargo with cDNA payload; once cDNA is inside the cell, this step is followed by the introduction of integrase enzyme, which mediates the integration of exogenous genome into the host cell. CRISPR-Cas-9 is a revolutionary therapeutic intervention; prior to the introduction of CRISPR-Cas-9, zinc-finger nucleases (ZFN) were the method of choice to edit DNA, but CRISPR-Cas-9 offers wide advantages, which range from increased efficacy to economization of designs [157]. The technique was developed in 2013, and it involves the use of Cas9 nuclease, which is guided by RNA sequence to the targeted site. On reaching the target site, the mutation sequence is removed, and the genome is repaired. Recently, a simpler version of CRISPR-Cas-9 has been developed, and this version uses the Cas-9 enzyme and RNA guide sequence (sgRNA), known as CRISPR-KO, without using a homology-directed repair pathway. CRISPR-KO has been designed to slice out the mutational region in the genome, hence resulting in the synthesis of indels in the host genome, and the technique is gaining attention for its clinical use in patients with sickle cell disorder [156].

### 4.6 Phytotherapeutics for ASD

According to scientific studies conducted over the years, numerous pharmacotherapeutic plant-based medications have been shown to be effective in treating psychiatric problems. Herbal pharmacological treatments could improve the core symptoms of autism with fewer side effects. Here, we have summarized several nutraceuticals that have antioxidant potential and which can subside autistic-like behaviors.

Polyphenols are known to have a beneficial effect on ASD. According to several studies, certain polyphenols, like resveratrol, control mitochondrial activity and guard against mitochondrial dysfunction, frequently found in people with ASD [196]. Several researches have shown that polyphenols, especially resveratrol, have preventive efficacy for patients with ASD [196]. It is important to mention here that polyphenols cannot cross the blood-brain barrier and may have low bioavailability in the brain, but the intestinal lumen contains them in abundance, and hence, have the ability to modulate the microbiota, which is of utmost importance in the pathogenesis of ASD [197].

The red, orange and yellow pigments present in plants, bacteria, or fungi that are fat-soluble are carotenoids [198]. They possess antioxidant and anti-inflammatory properties in multiple diseases [198-200]. Krajcovicova-Kudlackova and colleagues reported that lycopene, a kind of carotenoid, was reduced in the blood of autistic patients [201]. Astaxanthin, another carotenoid, also exhibits a neuroprotective role in cognitive function [202, 203]. Astaxanthin has also been demonstrated to alleviate behavioral issues and oxidative stress in the treated animals in a prenatal valproic acid (VPA)-induced mouse model of ASD [204]. In VPA-induced mouse model, green tea (*Camellia sinensis*) extracts, when administered from postnatal day 14 to day 40 in mice, showed significant improvement in ASD-like behavior [205]. Piperine was also tested in the same model and has prevented behavioral abnormalities and antioxidant markers [206]. For piperine, clinical studies have been started in ASD children to test its efficacy [207].

A number of systemic processes may interact to regulate mitochondrial function in ASD, including circadian and gut microbiome/permeability-derived products. Recent work indicates that variations in gut bacteria production of short-chain fatty acids (butyrate, acetate, and propionate) are significant factors in the regulation of mitochondrial function. Butyrate optimizes mitochondrial function, with effects that involve an increase in mitochondria-located sirtuins-3, which deacetylates and disinhibits the pyruvate dehydrogenase complex (PDC), leading to an increase in the conversion of pyruvate to acetyl-CoA, which increases ATP production by the tricarboxylic acid (TCA) cycle and oxidative phosphorylation [208]. It also needs to be noticed that acetyl-CoA is a necessary co-substrate for the conversion of serotonin to N-acetylserotonin (NAS) by aralkylamine N-acetyltransferase (AANAT); NAS is then converted by acetylserotonin methyltransferase (ASMT) to melatonin. The hyperserotonemia in ASD [209] may, therefore, arise from a decrease in the conversion of serotonin down the melatonergic pathway [210]. As most melatonin is produced within mitochondria, a decreased capacity to upregulate the melatonergic pathway is proposed to contribute to the mitochondrial dysfunction evident in ASD [211], as well as in many other medical conditions. This is relevant to both gut microbiome and circadian disruption in ASD, given that butyrate mediates its beneficial effects partly *via* the upregulation of the melatonergic pathway [212], while many of the impacts of pineal-derived melatonin over the circadian rhythm arise from pineal (or exogenous) melatonin, upregulating the mitochondrial melatonergic pathway [213]. Consequently, alterations in wider systemic processes (circadian and gut microbiome) may be intimately linked to variations in mitochondrial function, which has been proposed to contribute to the pathogenesis of ASD in early development *via* gut-amygdala effects that impact wider co-ordinated brain development [214, 210]. This also provides a frame of reference for wider epigenetic data on the alterations in microRNAs in ASD, with some of these miRNAs negatively regulate the capacity to induce the melatonergic pathway, as shown in ASD platelets, neurons, and intestinal epithelial cells [215]. Consequently, the regulation of mitochondrial function over the course of ASD pathogenesis and pathophysiology may be intimately linked to alterations in the interactions of the serotonergic and melatonergic pathways.

## CONCLUSION

There appears to be clear evidence that mitochondria are involved in disease; however, the risk factors for these mitochondrial dysfunctions need to be established and well understood. Recently, researchers have found novel mitochondrial abnormalities involved in the pathophysiology of ASD yet to be understood clearly and other genetic abnormalities, which are still not clear. Several preclinical and limited numbers of clinical studies have indicated that mitochondria can be a potential and robust target for the amelioration of clinical symptoms of ASD. Clinically, ASD constitutes a severe syndrome that poses great difficulty to the clinician in treating the disease; hence, it warrants a search for novel therapeutic interventions. In light of the earlier studies, the current review supports the pivotal role of mitochondria dysfunction, ranging from mitochondrial genomic mutation to oxidative free radicals generated in the mitochondrial microenvironment. Almost more than 5% of children with ASD were found to have well-defined mitochondrial disorders, while the remaining 10% of children were found to have some amount of other mitochondrial diseases. So, the disease needs to be addressed on two fronts: (i) how are mitochondrial dysfunctions related to ASD, and what are the various pathological pathways involved in the pathogenesis of ASD? (ii) To what extent can these mitochondrial pathological hotspots be effectively targeted by various pharmacological agents? With the advent of the genomic era, various genomic determinants of the disease have been identified. Owing to the genetic component involved in ASD, targeting these pathological genetic components offers an alternative therapeutic option. Further in-depth studies on the role of mitochondria in the pathogenesis of autism are warranted to identify the potential hotspots of the disease and, henceforth, effective targeting of these pathways.

## Figures and Tables

**Table 1:** Mitochondrial dysfunction and role of various mitochondrial ultra-structural dysfunctional frameworks, pro-inflammation, mutations and inefficiencies of enzymes in disease progression.

**Mitochondrial Dysfunction**	**Type of Study ** **(No. of Cases (n))**	**References**
Mitochondrial metabolites were significantly altered in ASD patients compared to healthy patients	Clinical (11)	[142]
Hyperlactacidemia	Preclinical	[174]
Significant reduction in levels of N-acetyl-aspartate1 (NAA) in diseased individuals compared to healthy individuals	Clinical (45)	[16]
Increased production of free radicals in mitochondria, increased lipid peroxidation, and depletion of reserve capacity of mitochondria	Preclinical	[170]
Over-expression of ETC complex IV proteins, which causes oxidative damage to mitochondrial micro-framework	Clinical (6)	[162]
Increased production of pro-inflammatory markers, like TNF-α, IL-1 and IL-1β	Preclinical	[125]
Deletion of mitochondrial genomic DNA at particular points	Clinical (10)	[178]
Increased incidences of mitochondrial genomic mutations triggered by oxidative imbalance	Preclinical	[170]
Significant reduction of ETC activity in the cerebellum of ASD patients	Clinical (8)	[107]
Increased levels of cyclic dipeptides (proline–phenylalanine), which triggers apoptosis.	Preclinical	[181]
Significant reduction in gene expression of ETC genes in the anterior cingulate gyrus	Clinical (10)	[171]
Increased levels of cathepsins, which cause increased expression of pro-inflammatory markers	Preclinical	[187]
Significant reduction in aconitase-5 activity	Clinical (15)	[175]
Variation in copy number of the mitochondrial genome	Preclinical	[193]
Significant reduction in expression of genes involved in synaptic structure and functioning	Clinical 69	[163]
Altered aspartate/glutamate carrier levels in patients with ASD	Preclinical	[192]
Significantly increased levels of phosphocreatine levels in ASD patients compared to control patients	Clinical (17)	[177]
Significant reduction in the efficacy of respiratory chain complex in ASD patients compared to healthy patients	Clinical (92)	[134]
Mitochondrial energy inefficiency	Clinical (67)	[178]

**Table 2:** Use of various therapeutic interventions for the treatment of autism with special emphasis on targeting mitochondrial pathogenetic hotspots involved in disease progression and side effects observed with their chronic use.

**Therapeutic Intervention**	**Biological Role**	**Side Effects**	**References**
Co-enzyme Q10/(Oxidized/ Reduced) Ubiquinone	Energy generation in the electron transport chain. Potent anti-oxidant usually targets free radicals generated in ETC within mitochondria	Gastro-intestinal disturbances	[122]
Ketogenic diet and modified Atkins diet	Acted on mitochondrial dysfunctional pathways and ameliorated the functioning of mitochondria	In the initial stage, diet was not well tolerated by the subjects	[41]
Niacin	Precursor of Nicotinamide adenine dinucleotide (NAD)	Dermatological allergy	[96, 128]
Methylcobalamin (B12) and Reduced folate (B9)	Promotes methylation/participates in the production of anti-oxidant enzymes	Excitability and sleep disturbances	[11,192]
Riboflavin	Flavin adenine dinucleotide (FAD)	Gastro-intestinal disturbances	[153]
Biotin (B7)	Co-factor for various enzymes involved in the metabolism of carbohydrates	None	[163]
Alpha-lipoic acid	Anti-oxidant/cofactor for various enzymes involved in cellular metabolism	Neurological disturbances	[137]
Leucovorin Calcium	Increases level of folate in nervous tissue	Hyper-excitability	[16]
Creatine monohydrate	Precursor of various cellular high-energy molecules	Nephrotoxicity	[164]
Thiamine (B1)	Involved in the TCA cycle	None	[159]
Pyridoxine (B6)	Cofactor for almost more than 40 enzymes involved in cellular metabolism	None	[173]
Vitamin C and Vitamin E	Decreases lipid peroxidation of biological membranes and helps to keep high reserves of anti-oxidants in cell	Hematological problems and gastrointestinal disturbances	[138]
N-acetyl-L-cysteine (NAC)	Precursor of various targeted anti-oxidant molecules	Gastrointestinal disturbances	[135]
acetyl-L-carnitine	Metabolism of long fatty acids/anti-oxidant	Gastrointestinal disturbances	[170]
Pantothenic acid (B5)	Cofactor for coenzyme A	Gastrointestinal disturbances	[138]

## LIST OF ABBREVIATIONS

ASD: Autism Spectrum Disorders
mtDNA: Mitochondrial DNA
ATP: Adenosine Triphosphate
PD: Parkinson’s Disease
ETC: Electron Transport Chain
ROS: Reactive Oxygen Species
CESLAC: Short and Long Acyl-carnitines
GDF15: Growth Differentiation Factor 15
FGF21: Fibroblast Growth Factor 21
LTA: Ligand Lipoteichoic Acid
LPS: Lipopolysaccharide
RNS: Reactive Nitrogen Species
GSH: Reduced Glutathione
GSSG: Oxidized Glutathione
SAM: S-adenosylmethionine
SAH: S-adenosyl-homocysteine
rRNA: Ribosomal RNA
tRNAs: Transfer RNAs
nDNA: Nuclear DNA
SNPs: Single Nucleotide Polymorphisms
AGC1: Aspartate/Glutamate Carrier
MELAS: Mitochondrial Encephalomyopathy, Lactic Acidosis, And Stroke-like Events
GWAS: Genomic-wide Association Study
CHARGE: Childhood Autism Risk from Genes and Environment
AMD: Atkins Modified Diet
SSADH: Succinic Semialdehyde Dehydrogenase
EEG: Electroencephalogram
MECP2: Methyl-CpG-binding Protein 2
mGluR5: Metabotropic Glutamate Receptor 5
nc-RNA: Non-coding RNA
ASO: Antisense Oligonucleotides
saRNAs: Small Activating RNAs
circRNA: Circular RNAs
ecDNA: Extrachromosomal DNA
ZFN: Zinc-finger Nucleases
